# *Babesia* sp. EU1 from Roe Deer and Transmission within *Ixodes ricinus*

**DOI:** 10.3201/eid1308.061560

**Published:** 2007-08

**Authors:** Sarah Bonnet, Maggy Jouglin, Monique L’Hostis, Alain Chauvin

**Affiliations:** * Institut National de la Recherche Agronomique, Nantes, France; † Ecole Nationale Vétérinaire de Nantes, Nantes, France

**Keywords:** Zoonosis, babesiosis, *Babesia* sp. EU1, Ixodes ricinus, roe deer, Capreolus capreolus, in vitro culture, sheep, dispatch

## Abstract

We report in vitro culture of zoonotic *Babesia* sp. EU1 from blood samples of roe deer in France. This study provides evidence of transovarial and transstadial transmission of the parasite within *Ixodes ricinus*, which suggests that this tick could be a vector and reservoir of EU1.

Babesiosis is a zoonosis caused by intraerythrocytic piroplasms of the genus *Babesia*, which are transmitted by ticks (*1*). In Europe, ≈30 human cases of babesiosis have been reported over the past 50 years and have been traditionally attributed to infection with the bovine parasite *B. divergens* transmitted by *Ixodes ricinus* (*2*,*3*). However, in 2003, Herwaldt et al. described the first molecular characterization of a new *Babesia* species, *Babesia* sp. EU1, isolated from 2 persons in Austria and Italy (*4*). Since this description, EU1 has been recovered from roe deer in Slovenia (*5*) and from *I. ricinus* in Slovenia (*6*) and Switzerland (*7*,*8*).

*Babesia* species EU1 merits increased attention as a potential agent of emerging tickborne disease in humans because its suspected vector, *I. ricinus*, is the most prevalent and widely distributed tick in Europe and frequently bites humans. To evaluate the public health importance of EU1, its vector, animal reservoir hosts, and geographic distribution must be identified. We identified EU1 in roe deer and in *I. ricinus* in western France.

## The Study

In January 2005 and January 2006, 89 roe deer at the Wild Fauna Reserve of Chizé (Deux Sèvres, France) were captured; blood was obtained through the jugular vein and analyzed for infection by *Babesia* spp. Parasites were isolated from autologous roe deer erythrocytes as described (*9*), except that 20% fetal calf serum (FCS) was used. Cultures were monitored for parasitemia by examination of Giemsa-stained thin blood smears. Parasites were then adapted to culture in blood from deer (*Dama dama)* from the Jardin des Plantes of Nantes (Loire Atlantique, France) and from sheep reared in our laboratory. Adaptation was conducted as described (*10*), except that 20% FCS was also used.

A total of 150 μL parasite genomic DNA was then prepared from 10-mL cultures (10% parasitemia, 2.5% hematocrit) according to the protocol of a commercial extraction kit (Promega, Madison, WI, USA) on merozoite preparations obtained by Percoll gradient centrifugation (Amersham, Uppsala, Sweden) with a density of 1.08 g/mL in 0.15 M NaCl. PCR amplifications were performed with 10 μL DNA and BAB primers (Table 1) as described (*11*). For positive samples, 5 μL DNA was subjected to a second amplification with EU1 primers (Table 1) as described by Hilpertshauser et al. (*8*), except that the annealing temperature was 63°C and uracil DNA glycosylase was not used.

**Table 1:** Nucleotide sequences of PCR primers used for amplification and sequencing of 18S rRNA genes of *Babesia* spp.*

Primer	Specificity	Sequence (5′→3′)	Annealing temperature, °C	Fragment size, bp	Reference
BAB	*Babesia*/*Theileria* spp.		60	359	(*11*)
GF2		GYYTTGTAATTGGAATGATGG			
GR2		CCAAAGACTTTGATTTCTCTC			
EU1	*Babesia* sp. EU1		63	362	(*8*)
Up		GTTTCTGMCCCATCAGCTTGAC			
Down		AGACAAGAGTCAATAACTCGATAAC			
CRYPTO	Apicomplexa		65	1,727	(*4*)
F		AACCTGGTTGATCCTGCCAGTAGTCAT			
R		GAATGATCCTTCCGCAGGTTCACCTAC			

*For parasites from tick samples, no sequence could be obtained with primer set CRYPTO because such primers likely hybridize to the *Ixodes ricinus* 18S rRNA gene and preferentially amplified this gene, probably because of its relative abundance.

During January 2005, 31 of 79 roe deer analyzed were infected with *Babesia* spp., as shown by parasite culture and PCR amplification with BAB primers. Of 29 cultures tested for EU1 with the corresponding primers, 59% were positive, which indicated an estimated global EU1 prevalence of 23% (Table 2). In January 2006, 5 of 10 cultures tested contained *Babesia* spp. parasites. Sequencing of the complete 18S rRNA gene from subcultures in autologous deer and sheep erythrocytes amplified with the primer set CRYPTO (Table 1) (*4*) showed that 2 of these cultures (C210 and C201) had 100% identity with the EU1 human strain (GenBank accession no. AY046575) (*4*). The unique sequence obtained has been deposited in GenBank (accession no. EF185818).

**Table 2:** PCR detection of *Babesia* sp. EU1 in blood samples from roe deer and in *Ixodes ricinus* larvae hatched from engorged females collected from roe deer*

Roe deer Identification	Primer BAB		Primer EU1	
	Cultures		Cultures	Larvae
C105	+		+	
C107	+		+	L107.1 (+)
				L107.2 (–)
C109	+		+	L109 (+)
C110	+		+	L110.2 (–)
C112	+		+	L112.1 (–)
				L112.2 (–)
C115	+		–	
C117	+		–	
C123	+		–	
C128	+		–	L128.3(+)
C129	+		+	
C139	+		+	
C151	+		+	L151 (+)
C156	+		+	
C162	+		+	
C163	+		+	
C164	+		–	L164 (–)
C167	+		+	
C171	+		+	L171.1 (–)
C172	+		–	
C176	+		–	L176.7 (–)
C177	+		–	L177.1 (–)
				L177.2 (–)
				L177.3 (+)
C179	+		–	L179.1 (+)
C180	+		+	
C185	+		–	
C188	+		–	
C193	+		–	
C169	+		+	
C189	+		+	
C157	+		+	
Total positive (%)	29		17 (58.62)	6/15 (40)

*+, positive amplification of a product of the expected size; –, no amplification.

In January 2005, a total of 106 engorged female adult *I*. *ricinus* were collected from the 31 roe deer harboring *Babesia* spp. Ticks were then reared in the laboratory at 22°C and a relative humidity of 80%–90%. Forty-two ticks (from 22 roe deer) laid eggs from which larvae were analyzed for parasites with BAB primers as described (*11*); 64% of larvae samples had a positive reaction. Amplification products from egg sample E177.3 and larva sample L177.3 that were sequenced showed 100% identity with the 18S rRNA gene of EU1 (*4*). The sequence has been submitted to GenBank (accession no. EF185819). Among positive samples, 6 of 15 analyzed for EU1 with specific primers showed a positive reaction (Table 2).

## Conclusions

Isolation of EU1 from roe deer in France confirms that these animals are reservoir hosts of the parasite and that EU1 is not restricted to 1 geographic area in Europe. A survey conducted in Slovenia showed that 21.6% of 51 roe deer tested were infected with EU1 (*5*) and a similar prevalence (23%) was observed.

To our knowledge, this is the first isolation of EU1 in culture in homologous erythrocytes and erythrocytes from other ruminants. Until now, *Babesia* sp. EU1 has only been detected in roe deer (*5*) and humans (*4*). It has also been detected in *I*. *ricinus* collected from sheep and goats in Switzerland (*8*); however, the ticks in that study may have acquired the infection at a preceding stage during a blood meal taken on another host.

In a study in Slovenia in 1997, 2.2% of 135 *I*. *ricinus* collected by flagging vegetation were positive by PCR for EU1 (*6*,*12*). PCR studies in Switzerland that examined ticks collected from domestic and wild ruminants with unknown parasitologic status showed that 1%–2% contained EU1 DNA (*7*,*8*). In our study, 40% of larvae samples from female ticks collected on *Babesia*-infected roe deer were infected with EU1. We assume that ticks do not necessarily become infected or transmit the parasite to the next generation after a blood meal on a EU1-infected host because 5 larval pools that originated from female ticks collected on EU1-infected roe deer were not infected (Table 2).

DNA sequences of the 18S rRNA gene were identical in parasites isolated from roe deer (C201 and C210) or *I*. *ricinus* samples. This finding indicates that deer and ticks were infected with the same organism, which may be transmitted by the tick. In addition to *I*. *ricinus,* EU1 DNA has been isolated from *Haemaphysalis punctata* ticks in Switzerland (*8*). However, during this survey, entire ticks or the apical part of fully engorged females were tested. Positive results from such samples indicated infection status only, not proof of the vectorial capacity of the tick (*11*).

We report that EU1 is transmitted within *I*. *ricinus* and that transovarial transmission occurs in this tick, as shown by detection of parasite DNA in eggs and larvae from females collected on roe deer. Some EU1-positive eggs and larvae can originate from adults engorged on EU1-uninfected roe deer, as observed in 3 roe deer (L128.3, L177.3, and L179.1). This finding suggests that the parasite was acquired during a preceding blood meal and that transstadial transmission occurred, at least from nymph to adult. Further investigations are needed to clarify the ability of *I*. *ricinus* to acquire and transmit *Babesia* sp. EU1. This species, which has been isolated from 2 human cases of babesiosis, should be studied to determine other potential reservoir hosts because of its potential as an emerging zoonotic pathogen.

## References

[1] Kjemtrup AM, Conrad PA. Human babesiosis: an emerging tick-borne disease. Int J Parasitol. 2000;30:1323–37. 10.1016/S0020-7519(00)00137-5 11113258

[2] Gorenflot A, Moubri K, Precigout E, Carcy B, Schetters TP. Human babesiosis. Ann Trop Med Parasitol. 1998;92:489–501. 10.1080/00034989859465 9683900

[3] Berry A, Morassin B, Kamar N, Magnaval JF. Clinical picture: human babesiosis. Lancet. 2001;357:341. 10.1016/S0140-6736(00)03640-0 11210995

[4] Herwaldt BL, Caccio S, Gherlinzoni F, Aspock H, Slemenda SB, Piccaluga P, Molecular characterization of a non-*Babesia divergens* organism causing zoonotic babesiosis in Europe. Emerg Infect Dis. 2003;9:942–8. 12967491 10.3201/eid0908.020748 PMC3020600

[5] Duh D, Petrovec M, Bidovec A, Avsic-Zupanc T. Cervids as *Babesiae* hosts, Slovenia. Emerg Infect Dis. 2005;11:1121–3. 16022795 10.3201/eid1107.040724 PMC3371785

[6] Duh D, Petrovec M, Avsic-Zupanc T. Molecular characterization of human pathogen *Babesia* EU1 in *Ixodes ricinus* ticks from Slovenia. J Parasitol. 2005;91:463–5. 10.1645/GE-394R 15986627

[7] Casati S, Sager H, Gern L, Piffaretti JC. Presence of potentially pathogenic *Babesia* sp. for human in *Ixodes ricinus* in Switzerland. Ann Agric Environ Med. 2006;13:65–70. 16841874

[8] Hilpertshauser H, Deplazes P, Schnyder M, Gern L, Mathis A. *Babesia* spp. identified by PCR in ticks collected from domestic and wild ruminants in southern Switzerland. Appl Environ Microbiol. 2006;72:6503–7. 10.1128/AEM.00823-06 17021198 PMC1610307

[9] Malandrin L, L’Hostis M, Chauvin A. Isolation of *Babesia divergens* from carrier cattle blood using in vitro culture. Vet Res. 2004;35:131–9. 10.1051/vetres:2003047 15099510

[10] Chauvin A, Valentin A, Malandrin L, L’Hostis M. Sheep as a new experimental host for *Babesia divergens.* Vet Res. 2002;33:429–33. 10.1051/vetres:2002029 12199370

[11] Bonnet S, Jouglin M, Malandrin L, Becker C, Agoulon A, L’Hostis M, Transstadial and transovarial persistence of *Babesia divergens* DNA in *Ixodes ricinus* ticks fed on infected blood in a new skin-feeding technique. Parasitology. 2007;134:197–207. 10.1017/S0031182006001545 17076925

[12] Duh D, Petrovec M, Avsic-Zupanc T. Diversity of *Babesia* infecting European sheep ticks (*Ixodes ricinus*). J Clin Microbiol. 2001;39:3395–7. 10.1128/JCM.39.9.3395-3397.2001 11526189 PMC88357

